# Using Social Psychology Principles to Develop Emotionally Intelligent Healthcare Leaders

**DOI:** 10.3389/fpsyg.2020.01917

**Published:** 2020-07-29

**Authors:** Neil E. Grunberg, John E. McManigle, Erin S. Barry

**Affiliations:** Department of Military and Emergency Medicine, Uniformed Services University of the Health Sciences, Bethesda, MD, United States

**Keywords:** social psychology, leadership, emotional intelligence, field theory, informal social communication, social comparison, cognitive dissonance

## Abstract

Healthcare providers must acquire extensive knowledge and skills to help promote physical health, behavioral health, and wellness; prevent and treat illnesses and injuries; encourage and guide rehabilitation; counsel and assist with decisions relevant to health, life, and death. In addition, 21st Century healthcare providers must develop leadership knowledge and skills to optimize their interactions and effectiveness with healthcare teams, patients, and patients’ significant others. Emotional intelligence is recognized as an essential component of leader education and development. It is important to optimally educate and develop healthcare providers with regard to components of emotional intelligence: self-awareness, self-regulation, social-awareness, and social regulation. Self-awareness focuses on understanding one’s own behaviors, cognitions, motivations, and emotions. Self-regulation emphasizes self-control and adaptability to various situations and settings. Social awareness includes understanding others’ behaviors, cognitions, motivations, and emotions. Social regulation draws upon the other components of emotional intelligence in order to optimize collaboration and cooperation and attainment of mutual goals with other people. The present paper presents four principles of Social Psychology that are relevant to developing emotionally intelligent healthcare leaders: Field Theory, Informal Social Communication, Social Comparison, and Cognitive Dissonance. Although these principles are well-established and have received extensive attention, analysis, and discussion in the academic social psychology literature, they are rarely mentioned in the emotional intelligence or leadership literatures. Therefore, each of these principles is briefly described in the present paper followed by an explanation of how each principle relates to the development of emotional intelligence in general and to emotionally intelligent healthcare leaders in particular.

## Introduction

Healthcare providers spend many years acquiring the knowledge and skills to help promote physical health, behavioral health, and wellness; prevent and treat illnesses and injuries; encourage and guide rehabilitation; counsel and assist with decisions relevant to health, life, and death. In addition to learning the vast amount of information relevant to perform various healthcare roles, 21st Century healthcare providers must develop leadership knowledge and skills to optimize their interactions and effectiveness with healthcare teams, patients, and patients’ significant others. Emotional intelligence is recognized as essential to leader education and development (Goleman, 1995, 1998, 2011, 2019). Therefore, it is important to optimally educate and develop healthcare providers with regard to the four elements of emotional and social intelligence: self-awareness, self-regulation, social-awareness, and social regulation (Goleman, 1998). Self-awareness focuses on understanding one’s own behaviors, cognitions, motivations and emotions. Self-regulation emphasizes self-control and adaptability to various situations and settings. Social awareness includes understanding others’ behaviors, cognitions, motivations, and emotions. Social regulation draws upon the components of emotional intelligence in order to optimize collaboration and cooperation and attainment of mutual goals with other people.

Understanding and increasing emotional intelligence have received an enormous amount of attention in the academic literature, popular press, self-help articles and books, and a wide variety of workshops focused on improving leadership, professional relations, and personal relationships (Goleman, 1995, 1998, 2011, 2019; Boyatzis and McKee, 2005). To complement these sources, the present paper presents four principles of social psychology relevant to developing emotionally intelligent healthcare leaders: Field Theory, Informal Social Communication, Social Comparison, and Cognitive Dissonance. Each of these social psychology principles has received extensive study and discussion in the academic psychology literature. Despite their relevance to emotional intelligence and leadership, these social psychology principles have not been widely considered or cited in those other literatures. To orient readers to these topics, definitions are first provided of psychology, social psychology, leadership, emotional intelligence, and healthcare practitioners. Then, each of the four social psychology principles is briefly described followed by an explanation of how they relate to emotional intelligence in general and to emotionally intelligent healthcare leaders in particular.

## Definitions

To orient readers to the topics discussed in this paper, we offer the following definitions of terms central to this paper:

• Psychology is the study of behaviors (actions), cognitions (perceptions, thoughts), motivations, and emotions (reasons underlying behaviors and cognitions).
• Social psychology is the study of behaviors, cognitions, motivations, and emotions in response to the actual, imagined, or implied presence of others.
• Leadership is influence on individuals and groups by enhancing behaviors, cognitions, motivations, and emotions to achieve goals that benefit the individuals and groups (Grunberg et al., 2018).
• Emotional intelligence includes self-awareness, self-regulation, social-awareness, and social regulation with regard to emotions and emotional information.

Self-awareness is knowing our own internal states, preferences, resources, and intuitions. Self-regulation is managing our internal states, impulses, and resources. Social awareness is awareness of other people’s feelings, needs, and concerns. Social regulation is adeptness relating to and inducing desirable responses with other people (Goleman, 1998).

• Healthcare practitioners include physicians, nurses, dentists, psychologists, physical and occupational therapists, and so on who serve as healthcare leaders by influencing the behaviors, cognitions, motivation, and emotions of other healthcare providers, students, administrators, patients, and patients’ significant others. To maximize effectiveness, healthcare leaders should become emotionally intelligent healthcare leaders who understand, apply, and practice components of emotional intelligence. Consideration, understanding, and application of key principles of social psychology are likely to help develop emotionally intelligent healthcare leaders.

## Social Psychology Principles Relevant to Emotional Intelligence

The four social psychology theories addressed in this paper received an enormous amount of scholarly attention in the 20th Century. They remain relevant today, yet they are rarely cited in the emotional intelligence or leadership literatures. They are presented below in chronological order of their appearance in the academic literature. That order of appearance occurred because they were built upon each other, conceptually and empirically. Their order of introduction into the scholarly literature also fits the “order” of the development of the four elements of emotional intelligence:

• Self-awareness: Field theory.
• Self-regulation: Field theory, Informal Social Communication.
• Social-awareness: Field theory, Informal Social Communication, and Social Comparison.
• Social regulation: Field theory, Informal Social Communication, Social Comparison, and Cognitive Dissonance.

## Field Theory

### Principle

Field Theory in Social Science (or “field theory”) describes behaviors, cognitions, motivations and emotions of each person as one’s “Life Space.” “Locomotions” are behaviors within one’s life space; “tensions” refer to thinking about (i.e., cognitions) or emotional pressures to act in a particular way; and “forces” connect tensions with locomotions. Field theory also describes how each person’s “life space” relates to those of other people with whom they interact (Lewin, 1936, 1951; Martin, 2003). Life spaces are dynamic in that they can change from moment to moment according to situations; a person’s current behaviors, cognitions, motivations, and emotions (their current life space) all are part of the situation.

### Background

Lewin (1936, 1951) believed that mathematizing each person’s behaviors, cognitions, motivations and emotions would help to understand individuals’ current psychology and help to predict future psychology. Life space includes the description of each person with regard to behaviors, cognitions, motivations and emotions. Personal goals or “goal regions” that are desirable and sought by the individual are labeled with “+” symbols. Situations that are undesirable are labeled with “−” symbols. Interestingly, Lewin and colleagues (Lewin and Lippitt, 1938) also studied leadership and proposed the first distinguishing types of leaders as autocratic, democratic, or laissez-faire.

### Relevance to Emotional Intelligence

Life space can be used to describe every psychological aspect of each person, including: behaviors (actions), cognitions (perceptions, thoughts, attitudes, and beliefs), motivations and emotions (why we act and think as we do); everything the person hopes to attain and to avoid; and where we “are” in our life space at a given time. As such, thoughtful reflection and identification of the goal regions within one’s life space, the influence of our emotions/motivations and thoughts, and “locomotions” (including actions/behaviors and thoughts) within one’s life space can serve to increase self-awareness – the first step in developing emotional intelligence.

### Relevance to Emotionally Intelligent Healthcare Leaders

Individuals who can accurately and comprehensively map out their life spaces, identify their positive regions (what they value and want to attain), identify their negative goal regions (what they want to avoid), know where they “are” in their life spaces, and understand the tensions and forces (motivations, emotions, cognitions, and situations) that are acting upon them within their life space will increase their self-awareness, thereby increasing their emotional intelligence. Using a life space analysis to increase one’s emotional intelligence will help healthcare leaders to recognize where they are with regard to their positive and negative goal regions (including values and concerns), set priorities, achieve personal balance, and know oneself (i.e., increase self-awareness) in ways that are likely to help with self-regulation, social awareness, and social regulations (i.e., the intersection of one’s life space with those of others, including colleagues and patients).

## Informal Social Communication

### Principle

According to informal social communication, unplanned or “informal” interactions and communication with other people influence behaviors and cognitions (e.g., beliefs, attitudes, and opinions), and the extent and types of interactions with others (or “functional distance”) are more important that actual physical distance (Festinger, 1950; Fay and Kline, 2011).

### Background

Informal social communication theory was inspired by field theory’s consideration of people influencing each other when their life spaces intersect (i.e., whenever they interact). Festinger, Schachter, and Back (Festinger et al., 1950) studied how people affected each other’s cognitions and behaviors based on the frequency and types of unplanned interactions in the real world. They reasoned the more frequently people interact and communicate with others whom they value, the more they will influence each other’s attitudes, opinions, beliefs, and behaviors. In fact, the more interactions that occurred, the more similar became attitudes and opinions among individuals who interacted. “Functional” space and distance (i.e., the extent to which people interact and communicate) rather than physical space and distance (i.e., geometric distance) dictate how frequently people interact and influence each other (Fay and Kline, 2011). For example, two people who live in houses that are physically close to each other but use private automobiles to travel and rarely walk in their neighborhoods will interact infrequently and, therefore, have large functional distance. In contrast, two people who live physically farther apart but who use the same bus stop on the same schedule will interact more frequently and, therefore, have close functional distance. As another example, people who interact frequently by video or audio communication devices while teleworking or for social interactions have close functional distance even though they may be hundreds or thousands of miles apart physically.

### Relevance to Emotional Intelligence

Informal social communication is relevant to the influence of interactions on behaviors, cognitions, motivations and emotions. Understanding that the frequency of interactions allows us to influence the cognitions and behaviors of others and, conversely, that frequency of interactions is directly related to how much we are influenced by others can increase our self-awareness and our self-regulation. Coupling informal social communication with field theory contribute to self-awareness and self-regulation. Knowing where we are in our life space and how others with whom we interact affect our individual psychology can help to enhance our emotional intelligence.

### Relevance to Emotionally Intelligent Healthcare Leaders

Many healthcare decisions are difficult to make because they involve consideration of an extensive amount of information, various opinions for the healthcare team members, and uncertainty. Healthcare leaders must consider each situation and the varied input they receive to make differential diagnoses, weigh treatment options, communicate with patients, and their significant others. To make the most thoughtful and well-reasoned decisions requires self-regulation of emotions. Emotionally intelligent healthcare leaders need to practice self-awareness and self-regulation. As discussed above, field theory provides a way to enhance self-awareness. Informal social communication highlights the influence others have on us depending on frequency and types of interactions. Emotionally intelligent healthcare leaders recognize that frequency of interactions and functional space contribute to the influence other people have on our behaviors, cognitions, and motivations. For example, those individuals with whom we have the most frequent contact and communication (purposely or informally) are likely to be particularly influential on our own beliefs, perspectives, and decisions that we make. Therefore, awareness and regulation of these influences are likely to influence the decisions and actions of emotionally intelligent healthcare leaders.

## Social Comparison

### Principle

According to social comparison theory, people need to judge the “goodness” of their behaviors, cognitions, and motivations/emotions. If physical realities are not available to make these judgments (e.g., when there are no unequivocal measures of correctness), then people compare themselves to other people to arrive at judgments about one’s own behaviors, cognitions, and motivations/emotions (Festinger, 1954; Dijkstra et al., 2010).

### Background

Festinger (1954) postulated that people have particular psychological needs, including the need to judge or evaluate their own behaviors and cognitions (perceptions, attitudes, beliefs, and opinions). If information is available that provides unequivocal information about a given behavior (e.g., Can I lift this weight?) or a given cognition (e.g., Is my answer to this mathematics problem correct?), then people are psychologically satisfied. If, instead, a physical reality or definitive and correct answer is not available, then people turn to “social reality” to evaluate their cognitions and behaviors. Social reality is based upon comparison with the behaviors and/or cognitions of people who we consider to be similar to us or, at times, with people who we admire or aspire to be.

### Applying Principle to Emotional Intelligence

Social comparison operates whenever a physical reality or unequivocally correct response is not available; that is, social comparison is a common and frequent psychological occurrence. Individuals who understand and are aware of social comparison occurring are attuned to the behaviors, cognitions, motivations, and emotions of other people and are, by definition, more adept at social awareness – the third “step” in developing emotional intelligence. Awareness of social comparison also helps to enhance self-awareness and self-regulation as we see and compare our behaviors, cognitions, motivations, and emotions with other comparable people.

### Relevance to Emotionally Intelligent Healthcare Leaders

Following from social comparison theory, opinions, attitudes, and behaviors of respected colleagues and role models are particularly influential when problems are complex and answers are not clear cut, as is often the case in healthcare. Healthcare also poses situations where upward social comparison operates as less experienced healthcare providers look to experienced healthcare providers for answers, advice, and guidance. Emotionally intelligent healthcare leaders who are sensitive to social comparison can help to create or encourage psychologically safety among the healthcare team members – i.e., interpersonal interactions where people feel accepted and respected and safe to learn, contribute, and challenge without fear of embarrassment or rejection (Edmondson, 1999; Appelbaum et al., 2016; Edmondson et al., 2016). In addition, emotionally intelligent healthcare leaders who understand social comparison can incorporate patient support groups that include people who have had or are having similar health challenges. Understanding social comparison theory along with field theory and informal social communication is likely to enhance self-awareness, self-regulation, and social awareness.

## Cognitive Dissonance

### Principle

Cognitive dissonance theory purports that people are most comfortable when behaviors and/or cognitions are consistent or “consonant” and are uncomfortable when behaviors and/or cognitions are inconsistent or “dissonant.” Moreover, individuals are likely to change their behaviors and/or cognitions (e.g., beliefs, attitudes, and perceptions) to achieve consonance (Festinger, 1957, 1962; Cooper, 2019).

### Background

Soon after proposing and examining social comparison theory, Festinger (1957, 1962) developed cognitive dissonance theory to explain why and how people respond when they become self-aware of conflicting behaviors and cognitions. He postulated that people are uncomfortable as they experience “cognitive dissonance” when they practice inconsistent behaviors, have inconsistent cognitions (e.g., perceptions, beliefs, attitudes, and ideas), or engage in behaviors and cognitions that are inconsistent with each other. To ameliorate or diminish this uncomfortable psychological state of cognitive dissonance, Festinger argued that people alter behaviors and/or cognitions to achieve or to perceive they achieve less dissonance (or cognitive consonance).

### Applying Principle to Emotional Intelligence

Cognitive dissonance theory indicates that people change cognitions and/or behaviors to achieve consonance within themselves. Consonance can be achieved by changing behaviors, cognitions, or both so that dissonance (i.e., any conflict or disturbing inconsistencies of behaviors, cognitions, or behaviors with cognitions) is reduced or eliminated. To determine whether consonance or dissonance exists, individuals must be aware of their behaviors, cognitions, motivations and emotions. It is also valuable to be aware of the behaviors, cognitions, motivations, and emotions of others with whom we interact and our relationships with those others. The psychological adaptations to reduce dissonance and to achieve consonance involve all four components of emotional intelligence: self-awareness and self-regulation with regard to within ourselves; social awareness and social regulation with regard to our interactions with others.

### Relevance to Emotionally Intelligent Healthcare Leaders

Many decisions and behaviors of healthcare practitioners become daily routine with experience. Yet, many decisions and behaviors of healthcare practitioners weigh on them because the “correct” answer is not always clear, easy, or an option. Also, conflicting opinions and options arise frequently based on input and preferences from healthcare teammates, patients, and patients’ significant others. As a result, healthcare practitioners often are confronted with cognitive dissonance resulting from varied input from oneself and from others. The healthcare leader who understands why and how this dissonance may bias decision-making and actions is more likely to be able to regulate the sources of conflict from self and others. The emotionally intelligent healthcare leader who is aware of the psychological tendency to reduce cognitive dissonance also may be more open to cognitive diversity (i.e., various perspectives and ideas from others and from oneself) before making decisions. The emotionally intelligent healthcare leader considers, understands, and applies the principles field theory, informal social communication, social comparison, and cognitive dissonance.

## Summary

Healthcare providers must master an enormous breadth and depth of role specific knowledge and skills depending on their particular areas and specialties of practice. In addition, healthcare providers serve as leaders who influence other healthcare professionals, patients, and patients’ significant others. To develop into optimally effective leaders, healthcare providers should develop awareness and regulation of their own and others’ behaviors, cognitions, motivations and emotions and become emotionally intelligent healthcare leaders. Understanding and applying the social psychology principles of Field Theory, Informal Social Communication, Social Comparison, and Cognitive Dissonance will help to develop emotionally intelligent healthcare leaders.

## Data Availability Statement

The original contributions presented in the study are included in the article/supplementary material, further inquiries can be directed to the corresponding author.

## Author Contributions

All authors made substantial contributions to conception, drafted the article or revised it critically for important intellectual content, and gave final approval of the version to be published.

## Disclaimer

The opinions and assertions contained herein are the sole ones of the authors and are not to be construed as reflecting the views of the Uniformed Services University of the Health Sciences or the Department of Defense.

## Conflict of Interest

The authors declare that the research was conducted in the absence of any commercial or financial relationships that could be construed as a potential conflict of interest. The handling editor declared a past collaboration with the authors.
